# Roots Form and Canals Morphology of Maxillary Second Premolar in a Sample of Yemeni Population

**DOI:** 10.1155/ijod/3380604

**Published:** 2025-02-27

**Authors:** AmatAlkhaliq M. Al-Sayaghi, Ahmed A. Madfa, Abdulbaset A. Mufadhal, Ibrahim Z. Al-shami, Ahlam M. Al-Shami

**Affiliations:** ^1^Department of Conservative Dentistry and Endodontics, Faculty of Dentistry, Sana'a University, Sana'a, Yemen; ^2^Department of Restorative Dental Science, Collage of Dentistry, University of Ha'il, Ha'il, Saudi Arabia; ^3^Department of Conservative Dentistry, Faculty of Dentistry, Thamar University, Dhamar, Yemen

**Keywords:** configuration, endodontics, maxillary second premolar, root canal morphology, Yemeni population

## Abstract

**Background:** The present study aimed to investigate the root form and canal anatomy of the permanent maxillary second premolar in a sample of Yemeni population using cone beam computed tomography (CBCT).

**Methods:** A total of 362 CBCT scans of maxillary second premolars of Yemeni patients aged between 15 and 60 years were analyzed to determine the anatomy of this tooth including number and form of roots, number of canals, number of orifices, and root canal configurations. Chi-square test was used to analyze the association between different variables.

**Results:** Of the 362 examined maxillary second premolars, 87.6% had one root, 12.1% had two roots, and 0.3% had three fused roots. Regarding the canal number, one canal was found in 181 teeth (50%), while two canals were found in 180 teeth (49.7%), three canals were found in one tooth (0.3%). One orifice was observed in 263 teeth (72.7%), two orifices in 98 teeth (27.1%), and three orifices were reported in one tooth (0.3%). Regarding root canal configuration, 98.6% of the sample were within the eight types of Vertucci classification. The majority of single-rooted second premolars (46.1%) exhibited type I canal configuration, type III found in 14.1%, and type II found in 9.9%. However, type IV found in 13.2% (48 teeth) of the sample, out of these, 88.6% (39 teeth) had two roots. Type VIII was observed in the three-rooted tooth (0.3%). Supplemental and additional canal types were found in 1.1% of the sample. Moreover, a new canal type was observed in 0.3% of the sample. According to Ahmed's coding system the most prevalent type was ^1^MSP ^1–1^ (46.1%) followed by ^1^MSP^1−2−1^ (4.1%), then type ^2^MSP B^1^ P^1^ (10.8%). Chi-square tests showed that the difference in root canal configurations among male and female was statistically significant.

**Conclusions:** Root and canal morphology of maxillary second premolars among the evaluated Yemeni population is highly variable and requires cautious evaluation prior to endodontic treatment. Majority of the sample were single-rooted teeth, most of them had a complicated and variable canal configuration. Moreover, significant gender disparities in internal and external morphology were observed.

## 1. Background

The root canal system exhibits a profound intricacy and considerable variety [1, 2], necessitating dental practitioners to possess knowledge of these variances. Insufficient expertise in this area raises the likelihood of overlooking necessary root canals and making mistakes during procedures, which can result in failure of endodontic treatment [3]. The endodontic treatment of maxillary second premolars poses significant challenges. The potential causes for this phenomenon are multifaceted, mostly encompassing aspects such as the quantity of roots, the quantity of canals, the orientation and longitudinal depressions of the roots, the diverse topologies of the pulp cavity, root curvature, and the challenges associated with radiographic visualization of the apical limit [4].

Research has demonstrated that there are main factors that contribute to the observed variances in the root canal system, such as ethnicity, age [5, 6], and gender [6, 7]. Vertucci conducted a study on the anatomical characteristics of the maxillary second premolar in patients from North America. The findings of his study revealed that the prevalence of a single canal with a solitary apex was 75%, while the occurrence of two canals at the apex was observed in 24% of the cases. Additionally, his study showed that the occurrence of three canals at the apex was observed in a mere 1% of cases [8]. According to a study conducted in China, the predominant canal design observed in teeth with two root canals was type IV (20.2%), followed by type II (16.3%), type III (11.4%), and type V (6.4%). According to the same study [9], it was observed that a solitary tooth possessed three root canals. Additionally, a study conducted by Abella et al. [10] examined the maxillary second premolar in the Spanish population. The findings revealed that 47.2% of single-rooted teeth displayed a type I canal shape, while type VIII canals were exclusively identified in three-rooted teeth. The examination of maxillary second premolars in the Saudi population indicated that majority of these teeth possess a single root 85.2%, with type I canal configuration being the most commonly observed (49.4%). According to this study, it was found that 80.2% of the subjects had a single apical canal, whereas 18.9% had two apical canals, and only 0.9% had three apical canals [11]. Furthermore, a research study conducted in Pakistan revealed that among a total of 115 maxillary second premolars, the majority (84%) had a single-rooted structure. The most common canal configuration observed was type I, accounting for 53.4% of the sample, followed by type II at 13.5% [12]. In the Brazilian context, it has been revealed that a single-rooted teeth has a higher prevalence than the two-rooted form. Specifically, the type I canal configuration was shown to be the most predominant, accounting for 49.9% of cases [13].

Several methods have been used for evaluating the root and root canal morphology of the human teeth among various populations including grinding or sectioning [14, 15], replication or modeling methods [16, 17], decalcification and clearing techniques [18, 19], radiography [20, 21], radiography with contrast media [22], and advanced computerized-aided techniques [23] such as microcomputed tomography (*µ*CT) [24, 25], and cone beam computed tomography (CBCT) [26–28].

CBCT is an imaging technique that provides high-quality, high-resolution, and accurate three-dimensional images of anatomic features which can be displayed in axial, sagittal, and coronal views. CBCT imaging shows high accuracy in root canal configuration identification, when compared with periapical radiograph which is inefficient to identify complex canal configurations [29]. In addition, it has the ability to decrease and eradicate the overlapping of nearby structures [30]. Furthermore, CBCT serves as a clinical tool that does not harm the tooth structure and reduces the time required for laboratory evaluations of root canal morphology. The benefits of lesser radiation exposure and the ability to do imaging in living organisms outweigh the advantages of using *µ*CT [31, 32]. Therefore, CBCT has been used widely to investigate the root form and canal configuration of human teeth in vivo. Due to the wide range of variations in the shape and number of roots, as well as the differences in root canal systems among various cultures and racial groups, it is important to comprehend this subject in order to provide appropriate endodontic treatment. Regretfully, there are currently no research in the literature on the root canal anatomy of Yemeni people's maxillary second premolars. Therefore, the present research aimed to describe the root and canal morphology of maxillary second premolar in a sample of Yemeni population using CBCT imaging.

## 2. Method

A retrospective cross-sectional observational study was conducted in a sample of Yemeni population resident in Sana'a to examine the anatomy of the root and canals in maxillary second premolars. The Medical Ethics Committee formally approved the College of Dentistry of Sana'a University compliance with medical ethics. During the period spanning from May 2020 to November 2022, images using CBCT technology were acquired. In considering the study's retrospective design, the ethics council of the College of Dentistry has granted an exemption for informed consent. During the course of this inquiry, the researchers made note of the gender and age. In order to protect the privacy of the patient, the information was maintained in a confidential manner. The participants included in the present investigation were individuals who had subjected to CBCT scanning for other diagnostic reasons.

The following equation was used to calculate the sample size (*n = z*^2^*. p* (*1−p*) */e*^2^); where *n* represents the desired sample size; *e* represents maximum size of standard error (was set at 5%); *z* represents number of standard deviations (1.96 for 95% confidence level); and *p* represents the estimated proportion of the outcome (i.e., estimated prevalence of two root canals) in the target population. Similar study in Saudi Arabia reported that 65% of studied maxillary second premolars showed two canals [33]. Therefore, this percentage was used as a reasonable estimate for two canal prevalence in Yemenis' maxillary second premolars.

  $$\begin{array}{c} n={\left(1.96\right)}^{2}\times \left(0.65\right)\times \left(1-0.65\right)/{\left(0.05\right)}^{2}=350\text{ teeth}. \end{array}$$

The experiment employed purposive nonprobability sampling to investigate a database including 2000 CBCT pictures. A total of 362 CBCT pictures were collected from Yemeni patients (180 males and 182 females), ranging in age from 15 to 60 years, who visited two different imaging centers located in different areas in Sana'a city. These centers are geographically located in two different and far distant areas where many dental clinics are located and to which many patients from Sana'a city and surrounding areas attend to get dental care. In addition, Sana'a (the capital city) contains a mixed population of more than three million from different governorates of the country. The inclusion criteria for this study encompassed high-quality CBCT images of fully developed maxillary second premolars of Yemeni individuals. The exclusion criteria included any image characteristics or dental work that could potentially cause distortion or diminish the quality of the image, thereby hindering its interpretation and analysis. These characteristics include teeth exhibiting advanced periodontal disease, deep caries, or extensive metallic restorations, as well as teeth that have been subjected to root canal treatment or crown restoration. Other exclusion criteria include teeth with open apices, resorption, fractures, calcification, developmental anomalies, or other pathologies. Additionally, images depicting adjacent implants, the presence of orthodontic treatment, or poor image quality were also excluded.

The evaluated CBCT images were captured using PaX-i3D Green imaging machine (VATECH Co., Ltd., Gyeonggi-do, Korea) following the recommended manufacturers' protocol. The acquisition parameters were as follows: 50–99 Kvp, 4–16 mA, 7.2–12 s exposure time, fields of view (FOV) of 5 × 5, 8 × 5, and 8 × 8 cm, with isotropic Voxel Size of 0.08–0.20 mm. CBCT pictures were subjected to analysis using Ez3D-i program (version Ewoosoft, Gyeonggi-do, Korea) in a 64-bit Windows 10 operating system. CBCT images were displayed in a Lenovo LCD screen featuring a resolution of 1280 × 1024 pixels, within the confines of a dimly lit environment. Using the software's picture editing features, tweaks were performed to improve the sharpness, brightness, and contrast of the photographs in order to maximize their visual integrity. Each of the three levels—the axial, coronal, and sagittal planes—was evaluated separately for each tooth. Initially, the analysis of external root morphology was conducted. Subsequently, the CBCT images were examined by sequentially navigating through the axial, coronal, and sagittal planes to ascertain the quantity and shape of the root(s). The internal morphology was assessed and the subsequent data were documented: quantity of root canals, number of orifices, and type of canal configuration based on both Vertucci's classification system, and Ahmed's coding system.

Before doing the evaluation, the examiner participated in calibration training. A random selection was made for 20% of the sample, which was thereafter reviewed separately by the examiner and an endodontist with more than 5 years' experience. By calculating the kappa coefficient, which came out to be 0.80, the degree of agreement between the two observers was evaluated. In order to reach a final consensus, the observers evaluated and discussed points of disagreement simultaneously. The same examiner performed a second analysis after the first review, but this time, the examiner was not aware of the earlier findings. The purpose of the second analysis was to evaluate the consistency of the examiner's observations. There was no statistically significant difference observed between the first and second observations.

The data analysis in this study involved the utilization of the Statistical Package for the Social Sciences (SPSS) software, version 23 developed by IBM Corporation. The analysis encompassed the examination of frequency distribution and cross-tabulation. The Chi-square and Fisher's exact tests were employed to assess the potential association between the gender of patients, and the morphology of their root and canal structures. The significance threshold was established at a level of 5% (*p* < 0.05).

## 3. Results

**Figure 1:**
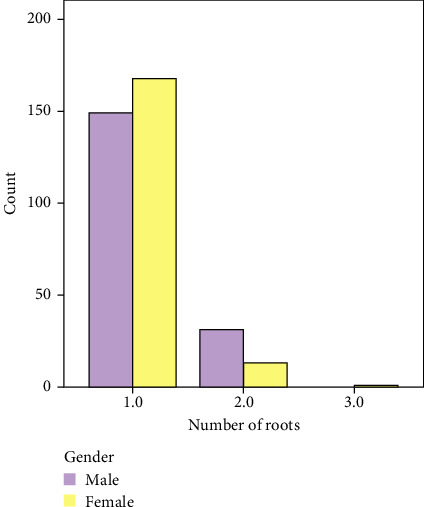
Percentage of each root group in relation to gender.

The distribution of number of roots in maxillary second premolar is shown in Table 1 and Figure 1. Among a total of 362 maxillary second premolars, 87.6% (317 teeth) possessed a single root. Notably, the proportion of females exhibiting this characteristic was higher, accounting for 92.3% (168 teeth), compared to 82.8% of males (149 teeth). The prevalence of teeth with two roots was found to be 12.1%, with a higher proportion observed in males 17.2% (31 teeth), compared to 7.1% (13 teeth) in females. Out of the entire sample size, a solitary tooth of female individual was observed to possess three fused roots, accounting for a mere 0.3% of the total.

**Figure 2:**
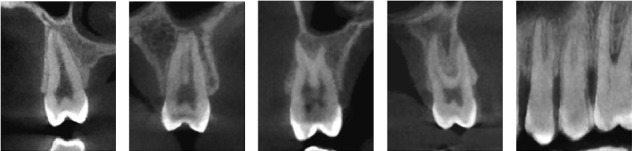
External root form of maxillary second premolar; (A) one root with one tip, (B) one root with two tips, (C) two fused roots, (D) two separated roots, and (E) three fused roots.

The current investigation demonstrates many external root configurations, as depicted in Table 2 and Figure 2. Out of a total of 317 teeth with a single root, 72.4% (262 teeth) exhibited single-tipped roots, whereas the remaining 15.2% (55 teeth) displayed double-tipped roots. Among the group of teeth with two roots, 5.8% (21 teeth) exhibited fusion of the roots, extending up to the apical one-third of the root. Additionally, 6.4% (23 teeth) displayed two distinct buccal and palatal roots. A mere 0.3% of the sample (one tooth) had three merged roots. There are a highly significant gender differences in the external root form with higher prevalences of one root with two tips and two fused root form among males.

**Figure 3:**
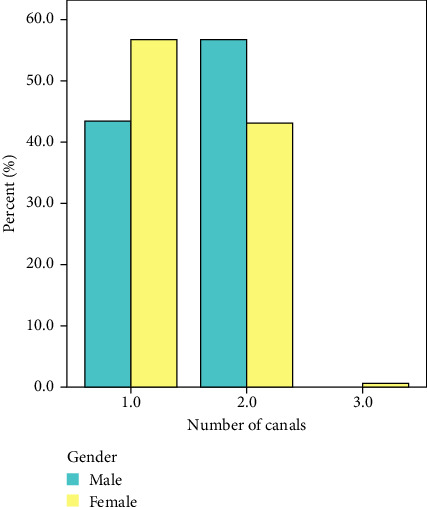
Percentage of canals number in relation to gender.

In the current investigation, the range of root canals observed varied from one to three canals, with a roughly equivalent distribution of about equal proportions for both single and double canals. Among the entire sample size of 362 teeth, it was observed that a single canal was present in 181 teeth (50%), while two canals were identified in 180 teeth (49.7%). Only one tooth exhibited the presence of three canals, accounting for 0.3% of the overall sample.  Table 3 and Figure 3 provide a more comprehensive breakdown of the number of canals in the maxillary second premolar, specifically in relation to gender. One canal was more in females (56.6%–103 teeth) than in male (43.3%–78 teeth). Two canals were more in males (56.7%–102 teeth) than in female (42.9%–78 teeth). The observed disparities between the two genders exhibited statistical significance (*p* < 0.05).

The current investigation provides a comprehensive analysis of the number of orifices present in the maxillary second premolar, as outlined in Table 4. Among the examined sample, it was observed that 263 teeth (72.7%) possessed a single aperture, whereas 98 teeth (27.1%) exhibited two orifices. The study documented the presence of three orifices in a single tooth, accounting for a mere 0.3% of the sample population. Based on statistical analysis, a notable disparity was seen between males and females in terms of quantity of orifices (*p* < 0.05). Specifically, the prevalence of a single orifice was shown to be greater in females compared to males.

**Figure 4:**
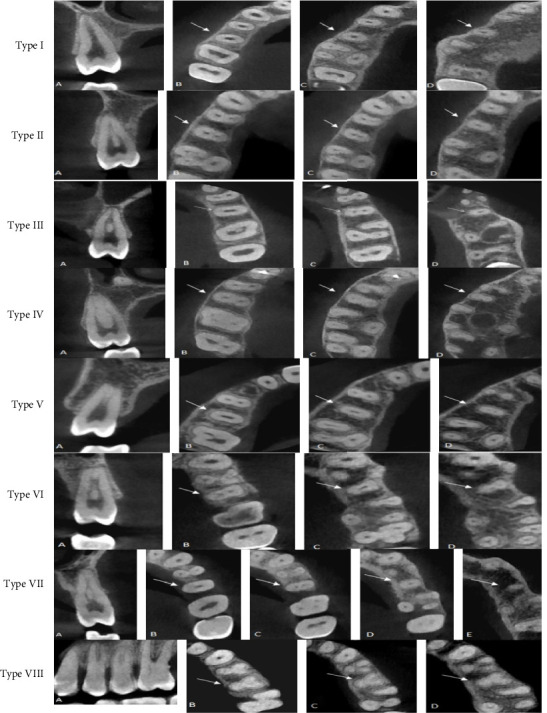
Various root canal types (at different cross-section from A–E) according to Vertucci's classification that found in the mesiobuccal root of maxillary second premolar.

**Figure 5:**
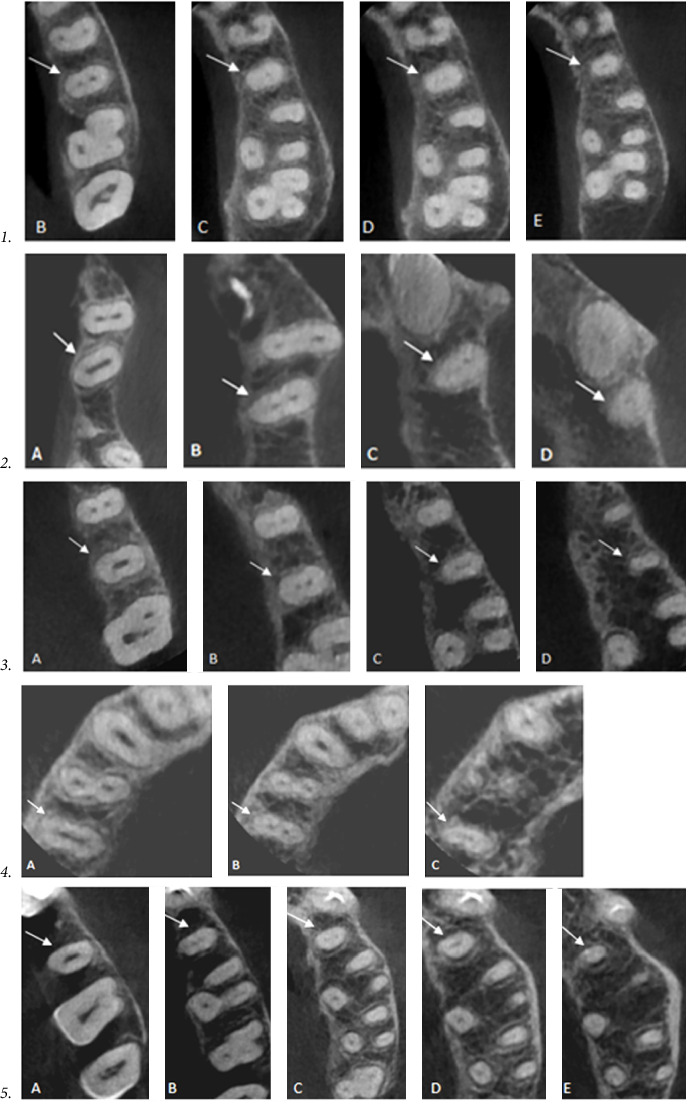
CBCT in the axial plane revealed supplemental and additional canal configurations (at different cross-section from A–E); **1**, Gulabivala supplemental canal-configuration type IV (2–1–2–1); Additional types; **2**, Sert and Bayirli type (1–2–3–1); **3**, Sert and Bayirli type (1–2–3–2); **4**, E. Senan type (1–2–3); **5**, New canal configuration type (1–3–2–1–2).

**Figure 6:**
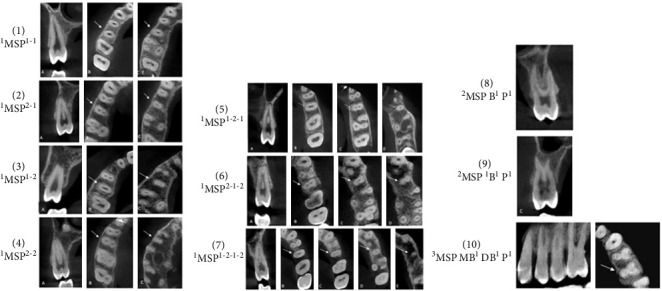
Single-rooted maxillary second premolar showing one code (1), two codes (2) (3) (4), three codes (5) (6), and four codes (7). Two-rooted maxillary second premolar showing one code (8), two codes (9). Three-rooted maxillary second premolar showing one code (10) according to Ahmes's coding system.

According to Vertucci's classification and other additional and supplemental types, the distribution of root canal configurations of maxillary second premolar among males and females is presented in Table 5. The findings indicate that a significant proportion of second premolars displayed a type I canal shape, accounting for 46.1% (167 teeth). This was followed by type III, which accounted for 14.1% of the sample (51 teeth), type IV was found in 13.2% (48 teeth). Additionally, type VIII was observed in a single three-rooted tooth, accounting for 0.3% of the sample (Figure 4). A supplemental and additional canal types were observed within 1.1% the sample (Figure 5 numbers 1–4). Furthermore, a novel form of canal was identified in 0.3% of the sample (Figure 5 number 5). There was a statistically significant difference in the variance of root canal designs between males and females. According to Ahmed's coding system, the form of ^1^MSP^1–1^ was the most prevalent (46.1%) followed by ^1^MSP^1−2−1^ (14.1%), then type ^2^MSP B^1^ P^1^ (10.8%) as shown in Table 6 and Figures 6.

## 4. Discussion

Numerous investigations have demonstrated that a variety of factors play a role in the variances observed in root canal morphology, such as ethnicity [34], age [5, 6], and gender [6, 7]. The present study used CBCT to investigate the exterior morphology and internal canal anatomy of the maxillary permanent second premolar within a sample of individuals from Yemen. The results indicate that a significant proportion of the teeth studied, specifically 87.6% (317 teeth), possess a single root. This observation aligns with previous studies conducted in different populations, including Brazil (90.3%) [7], Spain (82.9%) [10], Saudi Arabia (85.2%) [11], and Pakistan (84%) [12], as presented in Table 7. Nevertheless, the aforementioned proportion exceeds the figures documented in previous studies conducted in Jordan (55.3%) [33], Saudi Arabia (67%) [33], China (50.3%) [36], and Brazil (49.9%) [13].

In the studied sample, it was observed that 12.1% (44 teeth) exhibited the presence of two roots. This conclusion aligns with previous studies conducted in Brazil (9.7%) [7] and Spain (15.5%) [10]. In contrast, the aforementioned proportion is comparatively lower when compared with the rates observed in Saudi Arabia (30%) [33] and Jordan (44.2%) [35]. A single tooth was observed to possess three roots, accounting for 0.3% of the total sample. This finding aligns with a previous study conducted in Jordan, which reported a prevalence of 0.46%. However, it is lower than the rates reported in studies conducted in Spain (1.6%) and Saudi Arabia (3%) as documented by references [10] and [33], respectively. Furthermore, the existence of three roots in the maxillary second premolar has been reported in two published case reports, one from Brazil [37] and another from India [38]. Gender-related variations in the number of roots in maxillary second premolars exhibit notable distinctions, with a higher prevalence of two-rooted teeth observed in males. These results align with the findings reported by de Lima et al. [13] in the Brazilian population. Nevertheless, these results are incongruent with the findings documented in the Saudi population [11] and the Spanish population [10].

The present investigation revealed that 50% of the sample population have a singular canal. This result was consistent with previous research conducted in Türkiye, where a study reported a percentage of 48.66% [39], and a more recent study reported a percentage of 59.7% [40]. Similarly, a study conducted in China reported 50.3% [36]. However, this particular discovery contradicted the findings of other studies that reported larger proportions of single canals. For instance, in North America, a study found that 75% of cases had single canals [8], while in Brazil, the rate was 67.3% [7]. Similarly, in India, the reported percentage was 64.1% [41], and in Saudi Arabia, it was 80.2% [11]. In contrast, previous research has documented lower prevalence rates of single canal occurrences, such as 18.4% in Nigeria [42], 13.8% in Jordan [35], and 30% in Saudi Arabia [33].

The prevalence of two canals in this study was found to be 49.7%, which is consistent with previous findings in the Turkish population. A previous study conducted by Kartal, Özçelik, and Cimilli [39] indicated a prevalence of 50.64%, while a more recent study by Ok et al. [40] found that two canals were present in 40% of the sample. The present discovery shown a higher prevalence compared to previous research conducted on North American [8], Brazilian [7], and Saudi populations [11], which reported percentages of two root canals at 24%, 32.4%, and 18.9% correspondingly. On the contrary, the prevalence observed in this study was comparatively lower than the published figures in the Nigerian population (81.6%) [42], Jordanian population (85.7%) [35], and Saudi population (65%) [33]. In the present study, it was observed that a tooth within the sample exhibited the presence of three root canals, accounting for ~0.3% of the total sample size. These findings align with the results reported by Sert and Bayirli [7] in a study conducted in Brazil and [40] in a study conducted in the Turkish population. Additional information's regarding canal counts reported in different populations are included in Table 8.

This study provided a clear and definitive explanation that the count of orifices observed in the maxillary second premolar did not correspond to the number of canals present. Among the entire sample, a significant proportion of maxillary second premolars had a single aperture (72.7%), whereas a smaller percentage of teeth displayed two orifices (27.1%). This observation contradicts the findings of a study conducted in Saudi Arabia [33], which reported the presence of a single aperture in 55% of teeth and two orifices in 45% of teeth. Three orifices were identified in 0.3% of the sample, which is consistent with the findings reported by Al-Ghananeem et al. [35] in the Jordanian population. There were statistically significant variations seen between males and females in terms of the number of orifices. Specifically, males showed a higher prevalence of maxillary second premolar with two orifices.

There are several classifications of root canal anatomy in the literature that have been used in anatomical studies. Recently, Ahmed et al. [1] formulated a universal coding system that is able to include any type of root and canal configurations. However, to facilitate the comparison process with other studies, we mostly relied on the Vertucci classification, which is widely recognized and extensively employed in research. This classification system encompasses eight distinct types of canal configurations. In addition, the Gulabivala classification, which encompasses seven additional classifications, was also employed. Any types that were not included in the aforementioned classes were categorized as additional types.

The investigation revealed a significant degree of variability in the root canal structure of the maxillary second premolar. In general, this study documented a total of 13 different canal types. All variations of the root canal configuration as described by Vertucci were observed in maxillary second premolars with a single root, with the exception of type VIII which was only detected in teeth with three roots. Type IV of Gulabivala was detected in a single tooth, whereas three other types were identified in three separate teeth. A new canal configuration, which has not been documented in prior published research, was discovered.

Out of the 362 maxillary second premolars that were examined, 167 teeth (46.1%) displayed Vertucci type I. This observation aligns with or closely resembles previous findings in various populations, such as North American population with a prevalence of 48% [8], Turkish population with a prevalence of 54.5% [40], Spanish population with a prevalence of 47.2% [10], Chinese population with a prevalence of 50.3% [36], Saudi population with a prevalence of 49.4% [11], Pakistani population with a prevalence of 53.4% [12], and Brazilian population with a prevalence of 49.9% [13]. Nevertheless, the aforementioned results exhibited a greater prevalence of Vertucci type I canal configuration compared to certain populations. For instance, lower prevalence reported in Indian population (29.2%) [41], Jordanian population (13.8%) [35], and Saudi population (17%) [33].

The study revealed that type II canal structure was observed in 9.9% of the participants, which aligns more closely with the results given by Nazeer, Khan, and Ghafoor [12] for the Pakistani population, where a prevalence of 13.5% was documented. According to the study conducted by Elnour, Khabeer, and AlShwaimi [33], the prevalence of the described condition in the Saudi population was found to be 7%. The prevalence rates observed in this study were comparatively lower than the rates reported in previous studies conducted in North American [8], Indian [41], and Jordanian populations [35] (22%, 33.6%, and 24.9%, respectively). The prevalence of type III in the sample was 14.1%, which surpasses the percentages documented by previous studies [8] and [33], which showed rates of 5% and 9%, respectively. Moreover, type IV was observed in 13.2% of the sample, a percentage that closely aligns with the findings reported in North American population [8]. However, the aforementioned percentage is comparatively lower than the figures given by Al-Ghananeem et al. [35] 60.8% [41], 31.1%, and [33] 23% in the populations of Jordan, India, and Saudi Arabia, respectively.

A single tooth, constituting 0.3% of the sample, exhibited Vertucci type VIII canal shape. This fraction is similar to the findings reported by Vertucci, Seelig, and Gillis [8] in North American population (1%) [35], in Jordanian population (0.46%), and [33] in Saudi population (0.4%). According to sources [43] and [7], it was found that 0.9% of the teeth examined had extra types. Specifically [43], claimed a prevalence of 0.3%, while [7] indicated a prevalence of 0.6. Compared to our finding, a novel canal configuration (1-3-2-1-2) was identified in a single tooth (0.3%), which, to the best of our knowledge, has not been previously documented in the published articles.  Table 9 presents a comprehensive analysis of the canal configurations seen in the maxillary second premolar of the Yemeni population, as compared to other populations, within the context of this study.

### 4.1. Clinical Implication

There are several reasons that make the findings of this study applicable to clinical practice. In order to perform successful root canal treatment, dental students, general practitioners, and endodontists should have comprehensive knowledge of the potential variations in root and canal anatomy found in maxillary second premolars of Yemeni patients. For instance, they should consider the possibility to find a second and third root and/or canal in these teeth. Additionally, they should know that the number of orifices in maxillary second premolars does not necessarily correspond to the number of canals present. Another important finding of these teeth is the highly variable canal configurations (13 different canal types were encountered) which stresses the importance of preoperative evaluation and requires specific considerations during mechanical instrumentation. Prior to performing any endodontic treatment, it is advisable to obtain at least two preoperative radiographs with different horizontal angulations which can aid in the identification of a variable root canal system and the presence of extra roots and/or canals. Moreover, with the availability of CBCT imaging technique, more complex and challenging root and canal anatomies can be identified easily. This understanding enables dental clinicians and endodontists to enhance their proficiency in navigating complicated canal configurations and minimizing procedural mishaps. Another implication relays in the reported anatomical differences between males and females which highlight the importance of tailored treatment strategies. Providers of endodontic therapy should take these gender-based differences into account during the treatment planning to optimize treatment outcomes.

### 4.2. Limitations and Recommendations

The current investigation possesses certain limitations that necessitate careful consideration. One of these lies in the reliance on data from only two imaging centers, which could be perceived as a methodological constraint. The forthcoming multicenter study's findings have the potential to reflect the characteristics of the broader community more accurately. In order to enhance the precision of instructing medical professionals regarding the anatomical characteristics of the maxillary second premolar in the Yemeni population, it is advisable to conduct a more comprehensive analysis of the intricate nature of maxillary second premolars morphology. Moreover, it is imperative to take into account the impact of patients' age on the morphology of root canals in future research endeavors. Furthermore, it is recommended to do this anatomical inquiry throughout a wide geographical area encompassing diverse ethnic traits, as this approach has the potential to yield more definitive findings. CBCT is a valuable method for obtaining accurate anatomical information. Nevertheless, the clinical usefulness of this technology is limited due to its high cost and the potentially harmful radiation dose it delivers. Therefore, in order to optimize the efficacy of endodontic treatment, it is imperative for the practitioner to possess a comprehensive understanding of the diverse morphological variations that might occur in the roots and canals.

## 5. Conclusions

Under limitations of the present study, it can be concluded that the findings indicate that a significant proportion of the evaluated maxillary second premolars exhibited a single root with a solitary apex. The prevalence of maxillary second premolars with two roots was found to be greater in males compared to females. In general, there was a symmetrical occurrence of one root canal and two root canals, with a decreased prevalence seen for three root canals. The morphology of root canals in maxillary second premolars among Yemenis exhibited significant variability, with the predominance of Vertucci type I canal configuration. Various canal configurations were seen in these teeth, with uneven distribution. Additionally, complex canal types were identified. Moreover, this study has identified a hitherto type of canal that was not documented in previous research.

## Figures and Tables

**Table 1:** Number of roots in maxillary second premolar.

Number of roots	Male		Female		Total	
	Frequency	%	Frequency	%	Frequency	%
One	149	82.8	168	92.3	317	87.6
Two	31	17.2	13	7.1	44	12.1
Three	0	0	1	0.5	1	0.3

Total	180	49.7	182	50.3	362	100

*x* ^2^	*p* < 0.05					

**Table 2:** External root form of maxillary second premolar.

Number of roots	Root form	Gender					
		Male		Female		Total	
		*N*	%	*N*	%	*N*	%
One root	One tip	112	62.2	150	82.4	262	72.4
	Two tips	37	20.6	18	9.9	55	15.2

Two roots	Fused	14	7.8	7	3.8	21	5.8
	Separated	17	9.4	6	3.3	23	6.4

Three roots	Fused	0	0	1	0.3	1	0.3
	Separated	0	0	0	0	0	0

Total	—	180	49.7	182	50.3	362	100

*x* ^2^	*p* < 0.001						

**Table 3:** Number of root canals of maxillary second premolar in relation to gender.

Gender	One canal		Two canals		Three canals		Total	
	*N*	%	*N*	%	*N*	%	*N*	%
Male	78	43.3	102	56.7	0	0	180	49.7
Female	103	56.6	78	42.9	1	0.5	182	50.3

Total	181	50	180	49.7	1	0.3	362	100

*x* ^2^	*p* < 0.05							

**Table 4:** Number of orifices in maxillary second premolar in relation to gender.

Gender	One orifice		Two orifices		Three orifices	
	*N*	%	*N*	%	*N*	%
Male	118	65.6	62	34.4	0	0
Female	145	79.7	36	19.8	1	0.5

Total	263	72.7	98	27.1	1	0.3

*x* ^2^	*p* < 0.05					

**Table 5:** Root canal configuration of maxillary second premolar among males and females.

Vertucci classification	Male	%	Female	%	Total	%
Type I (1-1)	71	39.4	96	52.7	167	46.1
Type II (2-1)	16	8.9	20	11.0	36	9.9
Type III (1-2-1)	22	12.2	29	15.9	51	14.1
Type IV (2-2)	33	18.3	15	8.2	48	13.2
Type V (1-2)	19	10.6	15	8.2	34	9.3
Type VI (2-1-2)	11	6.1	0	0	11	3.0
Type VII (1-2-1-2)	6	3.3	3	1.6	9	2.5
Type VIII (3-3)	0	0	1	0.5	1	0.3
Gulabivala types						
Type IV (2-1-2-1)	0	0	1	0.5	1	0.3
Additional types						
Sert and Bayirli (1-2-3-1)	0	0	1	0.5	1	0.3
Sert and Bayirli (1-2-3-2)	0	0	1	0.5	1	0.3
E. senan (1-2-3)	1	0.6	0	0	1	0.3
New type						
(1-3-2-1-2)	1	0.6	0	0	1	0.3
Total	180	49.7	182	50.3	1	100
*x* ^2^	*p* < 0.05					

**Table 6:** Root canal configuration according to Ahmed et al. classification.

Codes	Male	%	Female	%	Total	%
^1^MSP^1–1^	71	39.4	96	52.7	167	46.1
^1^MSP^1−2−1^	22	12.2	29	15.9	51	14.1
^1^MSP^2–1^	16	8.9	20	11.0	36	9.9
^1^MSP^1–2^	15	10.1	14	7.7	29	9.1
^1^MSP^2−1−2^	11	6.1	0	0	11	3.0
^1^MSP^2–2^	6	3.3	3	1.6	9	2.5
^1^MSP^1−2−1−2^	6	3.3	3	1.6	9	2.5
^1^MSP^2−1−2−1^	0	0	1	0.5	1	0.3
^1^MSP^1−2−3−1^	0	0	1	0.5	1	0.3
^1^MSP^1−2−3−2^	0	0	1	0.5	1	0.3
^1^MSP^1−2−3^	1	0.6	0	0	1	0.3
^1^MSP^1−3−2−1−2^	1	0.6	0	0	1	0.3
^2^MSP B^1^ P^1^	27	15	12	6.5	39	10.8
^2^MSP^1^ B^1^ P^1^	4	2.2	1	0.5	5	1.4
^3^MSP MB^1^ DB^1^ P^1^	0	0	1	0.5	1	0.3

Total	180	49.7	182	50.3	362	100

*x* ^2^	*p* < 0.05					

**Table 7:** Studies of the number of roots of the maxillary second premolar in different populations.

Author	Year	Population	Sample size	One root %	Two roots %	Three roots %
Pécora et al. [4]	1993	Brazilian	435	90.3	9.7	—
Al-Ghananeem et al. [35]	2014	Jordanian	217	55.3	44.2	0.46
Elnour, Khabeer, and AlShwaimi [33]	2016	Saudi	100	67	30	3
Abella et al. [10]	2015	Spanish	374	82.9	15.5	1.6
Li et al. [36]	2018	Chinese	1403	96.2	3.8	—
Alqedairi et al. [11]	2018	Saudi	318	85.2	14.5	0.3
Nazeer, Khan, and Ghafoor [12]	2018	Pakistani	115	84	15.7	—
de Lima et al. [13]	2019	Brazilian	503	71.2	28.4	0.4
The current study	2021	Yemeni	362	87.6	12.1	0.3

**Table 8:** Studies of the number of root canals in maxillary second premolar in different populations.

Author	Year	Population	Sample size	One canal %	Two canals %	Three canals %
Vertucci, Seelig, and Gillis [8]	1974	American	200	75	24	1
Pécora et al. [4]	1993	Brazilian	300	67.3	32.4	0.3
Kartal, Ozcelik, and Cimilli [39]	1998	Turkish	300	48.66	50.64	0.66
Oginni [42]	2004	Nigerian	—	18.4	81.6	—
Jayasimha and Mylswamy [41]	2010	Indian	200	64.1	35.4	—
Ok et al. [40]	2014	Turkish	1301	59.7	40	0.30
Al-Ghananeem et al. [35]	2014	Jordanian	217	13.8	85.7	0.46
Elnour, Khabeer, and AlShwaimi [33]	2016	Saudi	100	30	65	5
Li et al. [36]	2018	Chinese	1403	50.3	49.7	—
Alqedairi et al. [11]	2018	Saudi	318	80.2	18.9	0.9
The current study	2021	Yemeni	362	50	49.7	0.3

**Table 9:** Studies of canal configuration of the maxillary second premolar in different populations.

Author	Year	Population	Type I	Type II	Type III		Type IV		Type V		Type VI		Type VII		Type VIII	Other
Vertucci, Seelig, and Gillis [8]	1974	American	48%	22%	5%		11%		6%		5%		2%		1%	—
Kartal, Ozcelik, and Cimilli [39]	1998	Turkish	48.6%	50.6%											0.66%	—
Jayasimha and Mylswamy [41]	2010	Indian	29.2%	33.6%		1.3%		31.1%		2.1%		1.2%		1%	1.3%	0.5%
Ok et al. [40]	2014	Turkish	54.5%	8.8%		3.6%		21.9%		10.8%		—		—	0.3%	—
Al-Ghananeem et al. [35]	2014	Jordanian	13.8%	24.9%		—		60.8%		—		—		—	0.46%	—
Abella et al. [10]	2015	Spanish	39.3%	22.5%		7.2%		19.8%		4.3%		3.2%		2.1%	1.6%	—
Elnour, Khabeer, and AlShwaimi [33]	2016	Saudi	17%	7%		9%		23%		23%		—		2%	0.4%	19%
Li et al. [36]	2018	Chinese	50.3%	10.4%		23.9%		5.9%		8%		0.3%		0.4%	—	0.7%
Alqedairi et al. [11]	2018	Saudi	49.4%	25.8%		5%		11.6%		5.7%		1.6%		—	0.9%	—
Nazeer, Khan, and Ghafoor [12]	2018	Pakistani	53.4%	13.5%		6%		3%		4.5%		12.8%		—	—	6.8%
de Lima et al. [13]	2019	Brazilian	49.9%	9.3%		2.2%		32.6%		4%		0.8%		0.8%	0.4%	—
The current study	202	Yemeni	46.1%	9.9%		14.1%		13.2%		9.3%		3.0%		2.5%	0.3%	1.5%

## Data Availability

The data are available on request from the authors.

## Nomenclature

CBCT: cone beam computed tomography
*µ*CT: microcomputed tomography.
